# Combined Influence of Lithium Nitrate and Metakaolin on the Reaction of Aggregate with Alkalis

**DOI:** 10.3390/ma16010382

**Published:** 2022-12-30

**Authors:** Justyna Zapała-Sławeta

**Affiliations:** Faculty of Civil Engineering and Architecture, Kielce University of Technology, Aleja Tysiąclecia Państwa Polskiego 7, 25-314 Kielce, Poland; jzapala@tu.kielce.pl

**Keywords:** alkali–silica reaction, pozzolan, lithium nitrate, synergistic effect, expansion, microstructure

## Abstract

The best known and effective methods for the reduction of the negative effects of an alkali–silica reaction in concrete include the application of mineral additives with an increased aluminium content and reduced share of calcium, as well as chemical admixtures in the form of lithium compounds. Because both aluminium and lithium ions increase the stability of reactive silica in the system with alkalis, it is possible to presume that the application of both corrosion inhibitors together will provide a synergistic effect in the ASR limitation. The paper presents the results of studies on the influence of combined application of metakaolin and lithium nitrate on the course of corrosion caused by the reaction of opal aggregate with alkalis. The potential synergistic effect was studied for the recommended amount of lithium nitrate, i.e., the Li/(Na + K) = 0.74 molar ratio and 5%, 10%, 15%, and 20% of cement mass replacements with metakaolin. The effectiveness of the applied solution was studied by measurements of mortars expansion in an accelerated test, by microstructure observations, and by determination of the ASR gels composition by means of SEM-EDS. The influence of metakaolin and the chemical admixture on the compressive and flexural strengths of mortars after 28 and 90 days of hardening were also analysed. The results of the studies revealed a synergistic effect for mixtures containing metakaolin at 15% and 20% cement replacement and lithium nitrate admixture in alkali–silica reaction expansion tests. It was found that corrosion processes in mortars with 5 and 10% levels of metakaolin became more severe after adding a lithium admixture to mortars with metakaolin only. The obtained results were confirmed by observations of the mortars’ microstructures. There was no synergistic impact of lithium nitrate and metakaolin on compressive strength characteristics. The compressive strength of mortars containing a combination of metakaolin and lithium nitrate decreased both after 28 and after 90 days, compared to mortars with metakaolin alone.

## 1. Introduction

The alkali–silica reaction (ASR) is an example of internal corrosion considered to be one of the reasons for the reduction of concrete structure durability. The damage caused by the reactivity of certain aggregate types in an alkaline environment was described for the first time by Stanton in the 1940s [1]. The silica reaction with sodium and potassium hydroxides in concrete pores gives, as a product, the sodium-potassium silicate gel of diversified calcium content, showing water absorption capability. The forming gel swells, causing a volumetric expansion of concrete, which results in the formation of fractures due to increasing tensile strength [2,3]. The aggregate reaction with alkalis resulted in serious damage to numerous road engineering structures and other concrete structures exposed to contact with moisture for a long time. Reports about the occurrence of a destructive reaction were published in numerous countries worldwide [4,5,6,7,8,9,10].

To reduce the risk of ASR occurrence, it is necessary to reduce or eliminate one of the factors that initiate it. The application of aggregates, which do not contain metastable forms of silica, eliminates the problem of alkaline corrosion entirely, but it is frequently difficult due to their limited availability. To prevent harmful expansion, being a measurable effect of ASR, it is recommended to use cements of reduced alkalis content. Stanton [1] showed that the reduction of alkalis content in cement below 0.6% is sufficient, while Obersholster proved that the dangerous expansion of concretes also occurs at lower alkalis contents in concrete [11]. In addition, the sources of alkalis in concrete, apart from cement, can also include aggregate, mineral additives, mixing water, and external sources, e.g., de-icing salts. As it is not clear what amount of alkalis from external sources is available for the ASR reaction, in the case of infrastructure elements, where medium or highly reactive aggregates were used, the reduction of the amount of alkalis in cement only and exclusively should not be treated as a sufficient method for protection against harmful ASR [12].

The use of mineral additives is mentioned, among other methods, for the reduction of ASR effects. The effectiveness of mineral additives in the reduction of destructive reaction of aggregate with alkalis depends on its activity, mineralogical composition of aggregate, alkalis content in cement, and amount of additive [13]. That means that, with a greater threat of destruction caused by the ASR, larger amounts of SCM should be used. As it is shown by numerous studies, the effectiveness of mineral additives goes down with a decreasing SiO_2_ content and an increasing CaO content in the additive, as well as with an increased alkalis content [14,15,16,17,18]. Therefore, metakaolin, fly ash with a small calcium content, granulated blast-furnace slag, and a silica powder are mentioned among the most effective. Mineral additives substantially reduce the alkalis content in the solution in concrete pores via the reduction of concrete permeability and removal of alkalis from the solution due to their binding by hydration products [15,16,18,19,20,21]. It is considered that the reduction mechanism of destructive ASR is also based on the limitation of availability of calcium ions from Ca(OH)_2_, which is consumed in the pozzolanic reaction [18,22]. However, the recent study shows that decreases of the pore solution pH in systems containing mineral additives are the reason for expansion reduction, due to the ASR, rather than the reduced calcium content [18,23].

In the ASR, a great role is assigned to aluminium ions. Mineral additives, which contain aluminium, reduce the expansion due to ASR more effectively, because aluminium shows the capability to reduce the solubility of reactive silica [24,25]. Aluminium ions transferring to the solution in pores are absorbed on the surface of silica, causing its passivation [24,26]. However, it has not been determined whether aluminium ions affect the composition and properties of ASR gels, thereby reducing their capability to swell.

Additionally, the application of lithium nitrate, in numerous cases, is considered effective in the elimination of the negative effects of ASR, both at the stage of concrete production and in elements, of which the durability was reduced as a result of ASR [27,28,29]. Although the role of lithium ions in the alkali–aggregate reaction was not clearly determined, it is assumed that lithium ions increase the silica stability in an alkaline environment, as well as modify the composition and swelling properties of reaction products [30,31]. It has been shown that the gels of compact texture and the reduced ratio of Ca/Si ions create in the presence of lithium ions. In the case of lithium nitrate, it is difficult to determine a simple relationship between the amount necessary for effective reduction of expansion due to ASR because of different effectiveness, irrespective of the degree of aggregate reactivity [32]. The effectiveness of lithium may also be reduced if the specific surface of reactive minerals in the aggregate increases [31]. However a [Li]/[Na + K] molar ratio of 0.74 for LiNO_3_ can sufficiently suppress the ASR-induced expansion of mortar/concrete containing most reactive aggregates [33,34,35].

Increasingly, a large numbers of papers have been published in the literature on the combination of potential ASR inhibitors to make them more effective against ASR. Afshinnia reported a synergy of fly ash with glass powder action in ASR mitigation, as compared with a smaller effectiveness of the fly ash only, while Shehata and Thomas showed the effectiveness of silica powder combination with fly ashes of various CaO contents [15,36]. The combination of granulated blast-furnace slag and fly ash with a high CaO content did not give the effect of strengthening, when comparing the obtained values with the value of expansion with the application of those two mineral additives separately. A slight increase in expansion was also shown with the combination of certain additives with each other, such as inter alia metakaolin with Class C fly ashes [37].

Only few studies are available on the effectiveness of simultaneous use of mineral additives and lithium compounds. The synergistic effect in the reduction of mortars with highly reactive aggregate expansion was visible with the use of a chemical admixture in the form of 0.55 M of lithium hydroxide and metakaolin [38]. The application of lithium nitrate in Li/Na + K = 0.56 molar ratio and replacing 30% of cement with addition of Class C fly ash (CaO = 27.7%) has proved to be ineffective in the protection of concrete against destruction, which was shown by Drimalas et al. [39]. The separate application of a mineral additive and lithium nitrate effectively reduced the expansion of concrete, with the reactive hornfels at a safe level. The authors have also shown a harmful effect of the applied system, resulting in increased expansion. Venkatanarayanan and Rangaraju have determined relationships between the chemical composition of fly ashes and the amount of lithium ions, expressed in the form of the [Li]/[Na + K] molar ratio, used to reduce the expansion of mortars with Spratt aggregate. It was shown that, with increasing contents of CaO, CaO_eq_, and CaO + MgO + SO_3_ in fly ashes, the amount of lithium required to reduce the expansion of mortars grows, while the increasing of SiO_2_, SiO_2eq_, and SiO_2_ + Al_2_O_3_ oxides share in fly ashes reduces this amount [40].

The paper presents the results of studies on the effectiveness of simultaneous application of lithium nitrate and metakaolin to reduce the effects of alkalis reaction with highly reactive opal aggregate. Previous studies by the author have shown that lithium nitrate applied in Li/Na + K = 1.0 molar ratio has shown high effectiveness, and the expansion of mortars in the accelerated test was reduced by 95% [41]. Additionally, the effectiveness of metakaolin applied in the amount of 15–20% of cement mass was shown, taking into account the effect of alkalis dissolution [42]. Because of the possibility of the deterioration of mortars with metakaolin workability [43,44,45] and the high costs of lithium admixture, it is justified to check the effectiveness of the action of smaller amounts of mineral additives and a limited amount of lithium ions. The effectiveness of metakaolin and lithium nitrate used alone and in combination were determined by an accelerated test method, according to a modified procedure of ASTM C1260 standard and ASTM C1567 standard [46,47]. Modification involved adding lithium nitrate to the immersion solution for samples with lithium nitrate alone and with an addition of lithium nitrate and metakaolin combined. Microstructures were observed under a scanning electron microscope and compositions of reaction products were determined.

## 2. Materials

### 2.1. Cement

Portland cement CEM I 42.5R was used in the tests. Its chemical composition is given in Table 1.

The alkalis content in cement, expressed in the form of sodium equivalent, calculated according to the relationship Na_2_Oeq = %Na_2_O + 0.658·%K_2_O, was 0.78.

### 2.2. Metakaolin

Metakaolin (Al_2_Si_2_O_7_) was obtained by kaolin calcining (T = 750 °C, t = 2 h). Table 1 presents the metakaolin composition. The grain size of metakaolin, determined by laser diffractometry, ranges from 0.18 to 14.6 µm. The median of grain size was determined for grains of 2.70 µm size [42]. Metakaolin was introduced to mortars, replacing 5–20% of cement mass. Table 1 presents the chemical composition of metakaolin (wt %).

### 2.3. Aggregate

Opal aggregate, originating from southern western regions of Poland, was used as a reactive aggregate. The tested aggregate was analysed from the petrographic point of view. Petrographic descriptions were made based on macroscopic and microscopic observations. Microscopic observations in transmitted light were carried out using an OLYMPUS BX51 polarisation microscope with a DP12 digital camera (Olympus, Tokyo, Japan). The studies were performed on standard microscopic preparations (thin plates), 35 × 20 mm in size. The carried out petrographic analysis of opal grains has shown that cristobalite–tridymite opal, CT opal is the basic component of the rock. Narrower zones built of crystalline proper chalcedony exist between zones built of opal. Small spaces between chalcedony zones are filled with fine-grained quartz. Opal reactivity is highest at a few percent of its content and grain size of 0.1 to 1.0 mm [48,49]. Opal with grain sizes of 0.5–1.0 mm, obtained by mechanical crushing of bigger rock fragments, was used in the tests. The percentage content of opal aggregate amounted to 6% of the total mass of aggregate. Non-reactive natural quartz sand, with a grain size of 0.125–4 mm, was used as the remaining aggregate for expansion tests. The same quartz sand, but with a grain size of 0.125–2 mm, was used in preparation the specimens for the strength activity test.

### 2.4. Lithium Nitrate Admixture

Lithium nitrate (LiNO_3_) was used in the form of a solution, by dissolving the solid form of analytical purity compound. Lithium nitrate was applied with the mixing water in the amount of 0.74 of molar ratio of lithium ions to the sum of sodium and potassium ions from cement.

### 2.5. Mixture Proportion

Table 2 shows the proportions of materials used to make mortars for the accelerated mortar bar test and mortar compressive and flexural strength tests. To evaluate ASR expansion, opal aggregate was used as a reactive aggregate, and non-reactive quartz sand was used as the remaining aggregate. Only quartz sand was used to evaluate the compressive and flexural strengths. The addition of metakaolin was introduced as a partial substitute for 5–20% of cement weight. The alkalis content in the binder was supplemented with addition of K_2_SO_4_, in order to preserve a constant amount of alkalis in the analysed systems. The w/s coefficient was kept on a constant level of 0.5 in mortars with a mineral additive, as well as with a mineral additive and chemical admixture, and w/c = 0.47 for the control and with lithium nitrate mortars. For the compressive and flexural strength test, the w/c = 0.5 for the control and with lithium nitrate mortars. Lithium nitrate was introduced in the form of a solution, retaining [Li]/[Na + K] ratio equal to 0.74.

## 3. Methods

### 3.1. Compressive and Flexural Strength Tests

The compressive and flexural strengths of the mortars were determined on 4 *×* 4 *×* 16 mm specimens. The specimens were made in accordance with the Polish standard PN-EN 196-1 [50]. All specimens from the given series, six in each series, were moulded and stored for 24h under laboratory conditions. After demoulding, they were stored for 28 and 90 days in water at room temperature.

The strength index was determined as the ratio of the compressive and flexural strengths of mortars, with the mineral additive to the control mortar after 28 and 90 days, according to EN 450-1 [51]. Higher activity indices are indicative of higher pozzolanic reactivity of additives used as partial replacements of cement.

### 3.2. Accelerated Mortar Bar Test

The aggregate reactivity level and the effectiveness of methods used for ASR mitigation were determined by measuring of mortars expansion, according to the guidelines of standards ASTM C1260 [46] and ASTM C1567 [47]. Accelerated ASR test methods are designed to provide an aggressive reaction environment, such as the immersion of mortar bars in 1M NaOH solution at 80 °C and affect mortars. In addition, mortars that contain lithium nitrate in their composition were immersed in the solution containing 1M NaOH and 0,74M LiNO_3_ in the same molar ratio as in mortars, i.e., 0.74.

Three 25 × 25 × 250 mm bars were made for each of the mortar series under analysis, in accordance with the requirements of Practice C305 [52]. The reference mortars and those containing potential corrosion inhibitors after moulding were stored for 24h above water at a temperature of 20 ± 2 °C and next for another 24h in water at 80 °C. After that time, a ‘zero’ measurement was made in a Graff–Kaufman apparatus, and then mortar bars were transferred to immersion solutions for 28 days. Lengths of samples were measured every day, until day 14 of the test, and then on days 21 and 28. In accordance with guidelines of standards ASTM C1260 and ASTM C1567, the expansion of mortars after 14 days, smaller than 0.1%, shows a minimum risk of destruction due to ASR, while expansion above 0.2% shows a high destruction risk. Expansion values between 0.1% and 0.2% show the need to carry out prolonged studies.

### 3.3. Scanning Microscopy Method

The microstructure of mortars was observed under a scanning electron microscope after 28 days of test. To this end, from each of analysed mortar series, one sample, with the dimensions 25 × 25 × 25 mm, was taken from the central part of the bar, and it was cut next to the size of 25 × 25 × 10 mm. Polished sections were made from the sample by grinding, followed by polishing on a diamond suspension. The studies were performed using a BSE detector under a scanning electron microscope coupled with an X-ray analyser in the micro-area (NOVA NANO SEM200 and Quanta FEG 205, FEI Company, Hillsboro, OR, USA). Chemical composition of ASR products from EDX analysis were based on averaged measurements taken from at least three locations within close proximity of each other.

## 4. Results and Discussion

### 4.1. Compresive and Flexural Strength Activity Index

**Figure 1:**
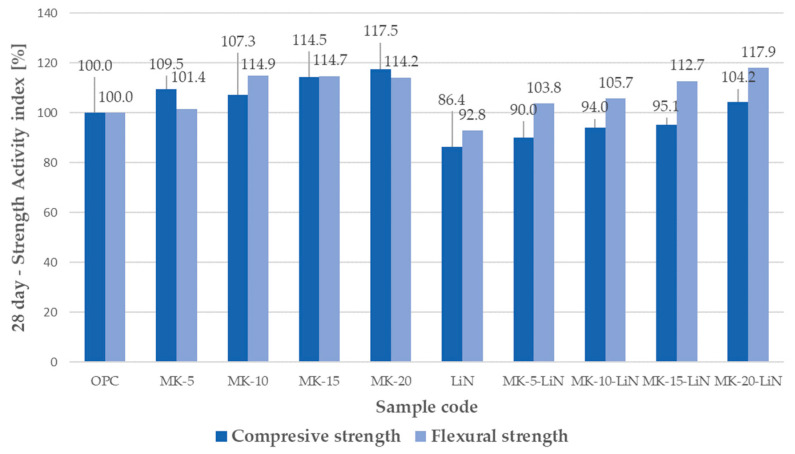
Strength activity index of mortars containing metakaolin and lithium nitrate at 28 days.

**Figure 2:**
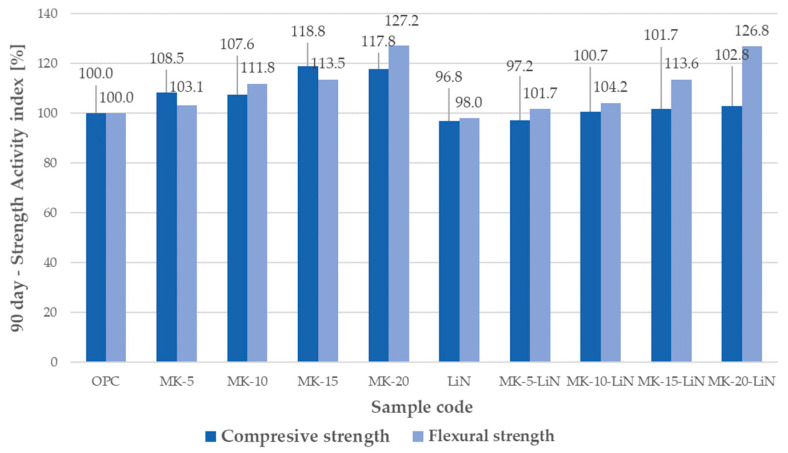
Strength activity index of mortars containing metakaolin and lithium nitrate at 90 days.

The effect of metakaolin, lithium admixture, and the combined use of metakaolin and lithium nitrate on the strength characteristics of mortars was evaluated by determining the compressive and flexural strength indices of mortars after 28 and 90 days of hardening. The results of the analyses are shown in Figure 1 and Figure 2.

As indicated in Figure 1, in mortars in which metakaolin was used as a partial replacement of cement, the mortar compressive strength improved, relative to the control mortars. At 28 days, the strength activity index was 107.3% for 10% metakaolin (MK-10) and 117.5% for 20% metakaolin (MK-20). In mortars with lithium nitrate (LiN) and the combined use of metakaolin and lithium nitrate, lower values of 28-days strength activity index were recorded. In the LiN series mortars, there was a reduction in the activity index to 86.4%. There was an approximate 10% decrease in the strength index for mortars with 5% metakaolin and lithium nitrate (MK-10-LiN) and a slight 4.2% increase in the activity index for 20% metakaolin and lithium nitrate (MK-20-LiN), compared to the controlmortars (OPC). The results indicate that lithium nitrate reduces the compressive strength of mortars, regardless of whether it is added separately as a mortar component or with a mineral additive. The strength of mortars cured for 90 days increased in all the series, as shown in Figure 2. In each case, the compressive strength increased significantly, relative to the 28-day strength, in mortars with 15% metakaolin (MK-15). Similar to the case of the 28-index, there was a reduction in the 90-day index in mortars with lithium nitrate (LiN) and metakaolin and lithium nitrate combined, compared to mortars with the mineral additive alone. For example, for mortars in the MK-15-LiN series, the value of the strength index decreased by about 17%, compared to MK-15 mortars, i.e., from 118.8% to 101.7%. This indicates that lithium nitrate lowers the compressive strength of mortars containing metakaolin added as a partial replacement of cement, also after 90 days of curing.

Compared to the reference mortar, there was an increase in the flexural strength of the samples with metakaolin both at 28 and 90 days. The strength results obtained were in line with literature data, indicating an improvement in the strength parameters of mortars with the addition of metakaolin [53,54,55]. However, the value of the 28-day strength activity index of mortars with lithium nitrate was 92.8%, which was lower than that of the control sample. In mortars with the combined use of metakaolin and lithium nitrate, the activity indices exceeded the values determined for control samples and were comparable to those calculated for mortars made with metakaolin alone. The strength activity index decreased only in mortars of the MK-10 and MK-10-LiN series from 114.9% to 105.7% at 28 days and from 111.8% to 104.2% at 90 days to the detriment of MK-10-LiN samples.

### 4.2. Expansion of Mortars

**Figure 3:**
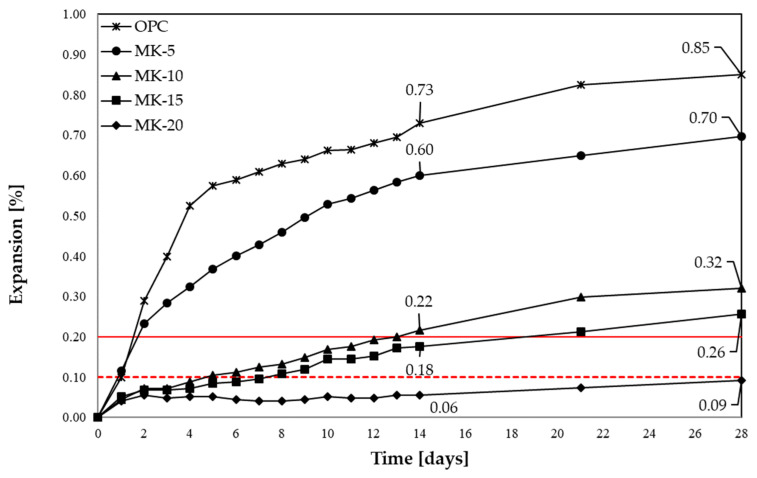
Linear changes of mortars with metakaolin in the accelerated method.

**Figure 4:**
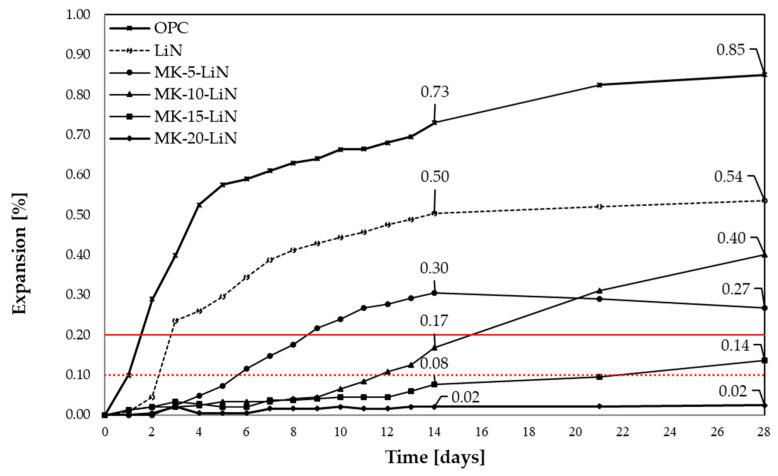
Linear changes of mortars with metakaolin and lithium nitrate in the accelerated method.

Figure 3 and Figure 4 present the results of linear changes of mortars with metakaolin and lithium nitrate, as well as with the combined application of metakaolin and lithium nitrate, analysed by the accelerated method in accordance with ASTM C1260 and ASTM C1567. In Figure 3 and Figure 4, the red lines represent the limit values of the expansion, indicating the presence of corrosion, according to the guidelines of standards ASTM C1260 and ASTM C1567.

Mortars with opal aggregate reached high expansion values after 14 and 28 days. Both the applied chemical admixture and metakaolin reduced the mortars expansion; however, they differed in effectiveness. The mortars expansion diminished with a growing amount of metakaolin, substituting for the Portland cement. The degree of aggregate reaction in mortars with metakaolin slowed down in the initial days of the test and depended on the percentage addition of the mineral additive. The reduction of expansion to a level that does not cause its degradation was observed for 20% substitution of metakaolin for cement. For samples with 10% content of metakaolin, a significant expansion growth was observed after 14 days. Lithium nitrate proved to be least effective, elongating the period of exceeding the reactivity limit of aggregate by only 1 day, as against the reference mortars.

The combined application of metakaolin and lithium nitrate resulted in the reduction of the expansion value in the analysed period of 14 days, as against the control mortars. It was shown that, from day 14, the increase in durability of mortars with opal aggregate occurred at 15% and 20% levels of metakaolin and with the added lithium admixture. In the case of samples with 5% content of the additive, a decrease of the expansion was recorded after 14 days, resulting from substantial cracking of mortars, causing a one-sided bend of two out of three bars. Comparing the obtained values of mortars expansion with linear changes of mortars with only lithium admixture or with metakaolin, it is possible to state that the effectiveness of simultaneous application of a mineral additive and chemical admixture is limited. Table 3 presents the relative reduction of expansion for the analysed mortars, as against the control sample (OPC).

As Table 3 shows, the synergistic effect of the simultaneous action of lithium and metakaolin was visible both for the 14-day and 28-day expansion for each of metakaolin contents, apart from 28-day expansion of mortars with 10% content of metakaolin and lithium nitrate. Two of mortar bars, with 5% content of metakaolin and lithium nitrate, were damaged after day 14 of the test; therefore, the results obtained after day 14 were lowered. Mortars, in which cement was replaced with 10–20% metakaolin, reached smaller expansions than the mortars with the lithium admixture. The obtained results suggest that, in order to achieve the synergy effect, it is necessary to apply minimum 10% of metakaolin and lithium admixture content. High expansion values of mortars with lithium nitrate and low expansion values of mortars with metakaolin indicate a higher potential of the mineral additive for ASR inhibition. In addition, considering the literature reports, the substitution of a higher metakaolin share for cement results in the deterioration of workability, and a combined application of additive and admixture could be considered favourable [43,44,45]. However, it is necessary to notice, that the strengthening effect is unstable because, in the period between day 14 and 28 of reaction, an increase in the expansion occurred, exceeding the linear elongation of mortars with the same 10% level of metakaolin.

It also should be emphasised that the combined application of the chemical admixture and additive could give better results if the alkalis content in the binder was not increased to a constant level. The effect of alkalis dilution in mortar samples would be then considered, which would also result in increasing the Li/(Na + K) molar ratio in the pore solution. The composition of the pore solution should be analysed to determine the Li/(Na + K) molar ratio. Wang showed that, even at an artificial increasing of the alkalis content in mortars with fly ash and simultaneous lithium nitrate addition, despite increased Na and K concentration in the pore solution, the [Li]/[Na + K] ratio was higher than planned. This improved the inhibitive properties of the used combination of the additive and chemical admixture [56].

### 4.3. Scanning Electron Microscopy

**Figure 5:**
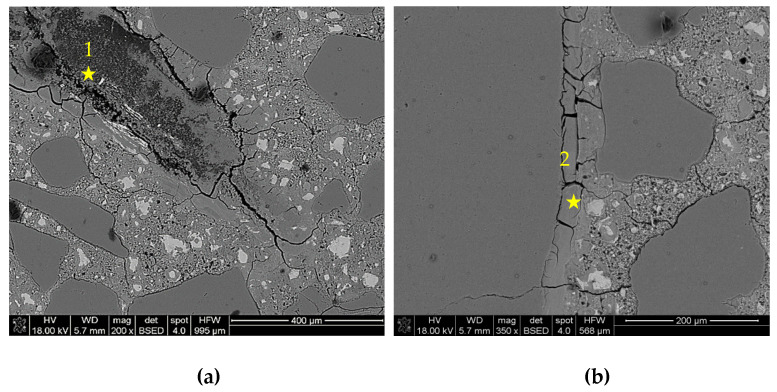
Microstructure images of control mortars: (**a**) gel in reactive aggregate; (**b**) gel located in ITZ. X-ray microanalysis at location 1 – 2 (see Table 4).

The microstructure was studied on polished sections of samples to determine the corrosion degree of reactive aggregate and cement mortar and to locate and determine the composition of ASR gels. Figure 5 shows the images obtained for the control mortar, i.e., without corrosion inhibitors, after 28 days of immersion in 1M NaOH solution. ASR products, which were formed in the volume of a reactive grain, exhibited swelling properties, resulting in cracking of the grain and surrounding it with cement paste. ASR gels also accumulated in the interfacial transition zone (ITZ). In the control samples, the destruction of the mortars was advanced, which was indicated by a large number of grains reacted with alkalis and significant expansion of the control mortars.

**Figure 6:**
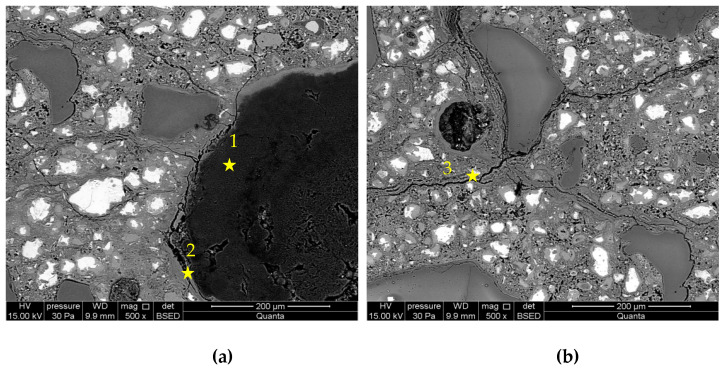
Microstructure images of mortars with lithium nitrate in a ratio of [Li]/[Na + K] = 0.74: (**a**) ASR gel in opal grain, (**b**) ASR gel in cement paste. X-ray microanalysis at location 1–3 (see Table 4).

Figure 6 shows the microstructure images of mortars with a lithium admixture, as analysed after 28 days of storage in the NaOH and LiNO_3_ solution. Despite the reduction of expansion by approx. 37%, a significant degree of reactive grains damage was found. Large amounts of ASR gels were visible both in the reactive grain and in the cement paste. Degraded grains of opal aggregate, from which numerous microcracks filled with reaction products were going away, were a characteristic image. Reactive grains in the BSE image featured a darker colour, which indicates the existence of compounds of smaller atomic mass. The thin reaction rim around aggregate was visible. In the analysed system, the lithium nitrate did not increase the stability of the reactive aggregate.

**Figure 7:**
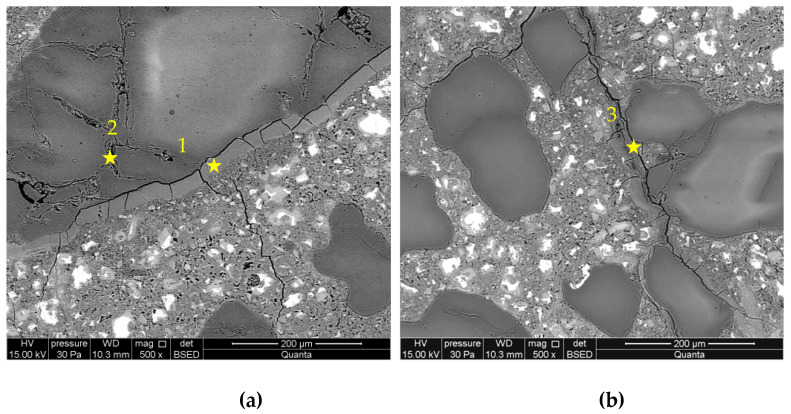
Microstructure images of mortars with 5% of metakaolin: (**a**) ASR gel in aggregate and ITZ; (**b**) ASR gel in cement paste. X-ray microanalysis at location 1 – 3 (see Table 4).

**Figure 8:**
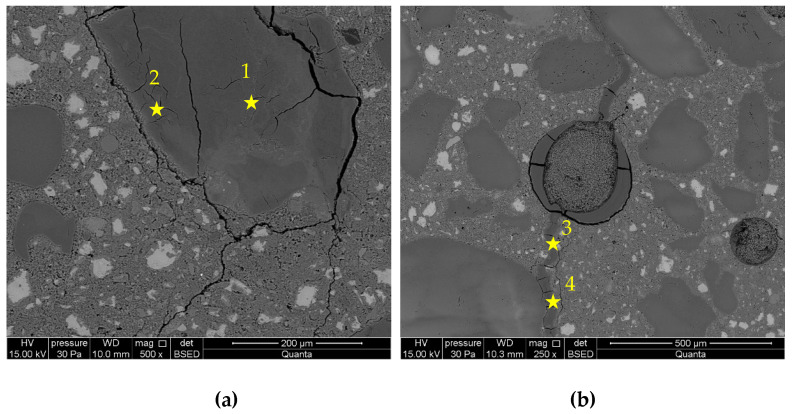
Microstructure images of mortars with10% of metakaolin: (**a**) ASR gel in aggregate; (**b**) ASR gel in cement paste. X-ray microanalysis at location 1 – 4 (see Table 4).

Figure 7 and Figure 8 presents the microstructure images of the mortars, in which cement was replaced with the addition of 5 and 10% of metakaolin. A smaller number of grains reacting with alkalis was observed, while the formed ASR gels, similar to in the control mortars, accumulated within the grain, in the interfacial transition zone, and in the cement matrix. However, the degree of grains degradation was smaller than in the control mortars and depended on the amount of introduced additive. The more metakaolin in the cement mortar, the smaller the number of reacting opal grains.

**Figure 9:**
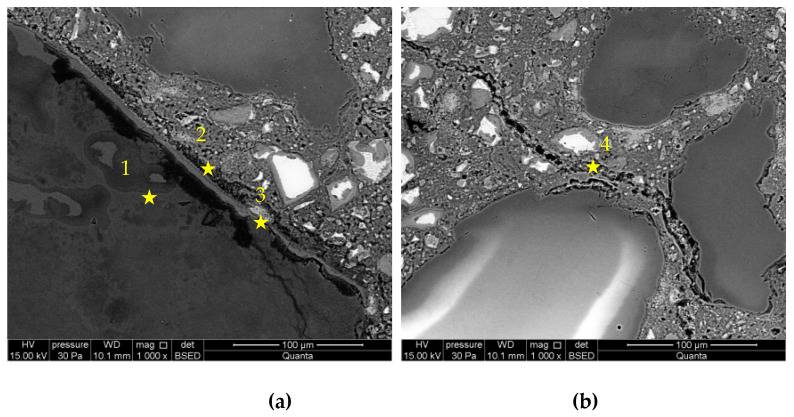
Microstructure images of mortars with metakaolin and lithium nitrate in 5% of metakaolin and [Li/Na + K] = 0.74 samples: (**a**) ASR gel in aggregate and ITZ; (**b**) ASR gel in cement paste. X-ray microanalysis at location 1 – 4 (see Table 4).

**Figure 10:**
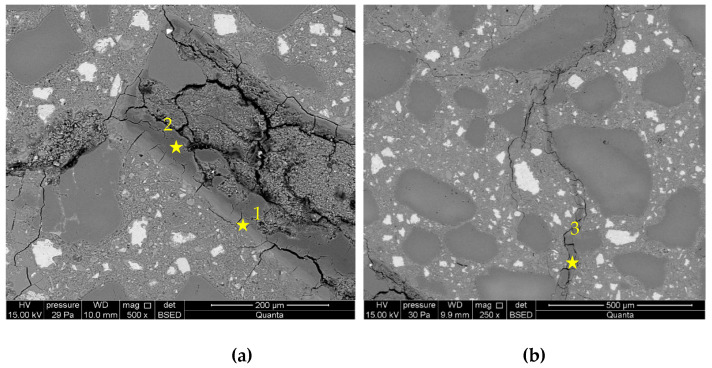
Microstructure images of mortars with metakaolin and lithium nitrate in 10% of metakaolin and [Li/Na + K] = 0.74 samples: (**a**) ASR gel in aggregate; (**b**) ASR gel in cement paste. X-ray microanalysis at location 1 – 3 (see Table 4).

Figure 9 and Figure 10 presents microstructure images of mortars with a combined addition of metakaolin and lithium nitrate. In mortars with 5% of metakaolin and lithium nitrate addition, a high degree of corrosion processes advancement was recorded, which confirms the intensified formation of ASR gels located within the grain and cracks propagating to the cement paste matrix. Reactive silica in the opal grain degrades, due to the influence of aggressive ions, to a greater degree than in the case in mortars with metakaolin. Microstructure images are closer to those recorded in mortars with lithium nitrate, which shows that the role of lithium nitrate is dominating. In the interfacial transition zone, compact gel accumulated, which was different from the gels observed in control mortars and those with metakaolin. Additionally, the microstructure of mortars with 10% of metakaolin and lithium nitrate shows that the applied system of inhibitors does not prevent the reaction between the aggregate and alkalis. Nevertheless, the gel visible in the cement paste formed in smaller amounts than in the control mortars and mortars with lithium nitrate. Microstructure images are similar to those observed in mortars with metakaolin only.

Table 4 presents chemical compositions of ASR gels in control mortars and in mortars with corrosion inhibitors. The composition was determined for gels formed within the grain, in ITZ, reaction rim around grain, and observed in the matrix of cement paste, which are indicated in Figure 5, Figure 6, Figure 7, Figure 8, Figure 9 and Figure 10.

The compositions of the alkali–silica reaction products varied, depending on the reaction degree and location [57,58]. ASR gels located within the grain had a different composition than those observed in the ITZ or in the matrix of the cement paste. The gels that accumulated in the matrix of cement paste contained more calcium than the gels formed in the reactive grain. The furthest was the gel located from the reactive grain, with the higher CaO contents and smaller SiO_2_ contents. This resulted in higher Ca/Si ratios in the reaction products, situated in the matrix of cement paste, rather than in the reactive grain. With the increasing content of metakaolin in the mortar, the Ca/Si ratio in gels in the cement paste decreased. In mortars with a 10% level of metakaolin and 10% content of metakaolin and lithium, the gels, despite their accumulation in the cement paste, featured small calcium contents. This is related to a smaller content of calcium ions in the solution in pores, resulting from the occurrence of pozzolanic reaction between Ca(OH)_2_ and metakaolin.

In the analysed mortars, the ITZ gels had different compositions. The different Ca/Si ratio in mortars resulted from the fact that the gels accumulated in the contact zone of the paste, with reactive and non-reactive grains. Gels, which were forming in the contact zone of the paste and reactive aggregate, or created the reaction rim, contained more calcium. While the gels that accumulated in the contact zone between non-reactive aggregate and cement paste showed smaller calcium contents, which was visible, in particular, in the mortars with 10% level of metakaolin. In the reactive grain in the control mortars, the formed products showed an increased Ca/Si ratio, as against the gels observed in mortars with lithium nitrate and in mortars with the addition of lithium nitrate and metakaolin combined. A high degree of corrosion process advancement in the grain was related to a local reduction of pore solution pH, which was favourable for the dissolution of calcium hydroxide. Calcium ions can diffuse to reaction sites and penetrate into reactive grains [59]. With the presence of mineral additives and lithium nitrate, the gels formed in the reactive grain had a lower Ca/Si ratio than in control mortars. The obtained results were consistent with the literature data, which indicated that products of smaller calcium content were formed in the presence of both metakaolin and lithium compounds, which reduced the swelling properties of ASR gels [60].

The results of analyses presented in Table 4 show that gels in mortars with corrosion inhibitors feature smaller values of (Na + K)/Si ratio, which results both from a smaller content of alkalis, mainly sodium, and a higher silica content. This indicates a smaller degree of reactive grain deterioration in mortars with metakaolin and with metakaolin and lithium nitrate combined against control samples. Based on the obtained compositions of gels in mortars with metakaolin and with metakaolin and lithium nitrate combined, it is not possible to clearly show what changes occur after the introduction of a chemical admixture. Instead, it was shown that, in the presence of lithium nitrate, the opal grains react with the alkalis, and products of reduced Ca/Si ratio are formed. In mortars with 5% of metakaolin and lithium nitrate, where the chemical admixture plays a dominating role, the Ca/Si ratio diminishes in products in grains, as well as the alkalis content. Hence, this confirms the literature reports indicating a reduced content of calcium ions in ASR gels. The increased amount of gels in the cement matrix probably resulted from the intensified dissolution of reactive silica in the grain and may be responsible for the increased expansion of the mortars. Wang recorded an increased concentration of sodium and potassium ions in the pores of mortars with fly-ash and a lithium admixture as against mortars without corrosion inhibitors, as well as mortars with lithium nitrate only, with a simultaneous increase in the Li/Na + K ratio [56]. Because of the limited research materials, the phenomenon requires further studies, including the determination of changes in the composition of the solution in mortar pores over time.

## 5. Conclusions

The main objective of the paper was to show the effectiveness of the simultaneous application of metakaolin and lithium nitrate admixture in the mitigation of the negative effects of opal aggregate reactivity. Based on the strength activity index result, the performed expansion tests and analyses of the mortars’ microstructures, together with the determination of the ASR gels’ compositions, the following has been found.

1. Partial replacement of cement with metakaolin improves the compressive and flexural strengths of mortars, relative to mortars without the mineral additive. The addition of metakaolin, combined with lithium nitrate, does not show a synergistic effect. Lithium nitrate reduces the compressive strength of mortars, as well, if used together with the mineral additive.
2. Expansion decreased to a safe level within 14 days in the mortars, in which more than 15% of the cement was replaced with metakaolin. In the period extended to 28 days, only 20% replacement of cement with the metakaolin addition protected the mortar against ASR-induced deterioration. Lithium nitrate turned out to be the least effective method for mitigating destructive ASR.
3. The combined use of metakaolin and lithium nitrate was effective, when lithium was applied together with 15% and 20% of metakaolin in the mortar. Only in these systems was the synergy between metakaolin and lithium admixture observed. The synergistic effect in mortars with 10% of metakaolin and lithium nitrate was unstable in the analysed period of time.
4. Based on the microstructure studies, the degradation level of control mortars, observed as the number of corrosion centres, substantially exceeded that of mortars with corrosion inhibitors. The degree of corrosion processes was confirmed by the obtained values of mortar expansion.
5. The image of opal grains degradation in mortars with lithium nitrate differed from those observed in the control mortars. Despite the observed high progress of grain corrosion processes, the gels formed inside it showed a lower Ca/Si ratio, as against the gels in the grains of control mortars.
6. A lithium admixture plays a dominating role in the mortars with 5% of metakaolin and lithium nitrate, which was indicated by microstructure images similar to the microstructure of mortars with the lithium admixture only. The combined application of both potential corrosion inhibitors was unfavourable in the studied system, which was also confirmed by the macroscopic degradation of the mortars after 14 days of reaction.

## Figures and Tables

**Table 1:** Chemical composition of cement and metakaolin.

Material	SiO_2_	Al_2_O_3_	Fe_2_O_3_	CaO	MgO	SO_3_	SrO	K_2_O	Na_2_O	TiO_2_	LOI	N.s.p
Cement	19.61	5.43	2.79	63.92	1.46	3.17	-	0.90	0.19	0.45	3.28	0.65
Metakaolin	55.80	41.06	0.73	0.09	0.10	-	0.11	0.92	0.00	0.69	0.31	-

LOI—loss of ignition, N.s.p –non-soluble parts in HCl and Na_2_CO_3_.

**Table 2:** Relative proportions of material used in preparation of mortar bars.

Sample Code	Cement Content [%]	Metakaolin Content [%]	[Li]/[Na + K]	Opal Aggregate [%]	Quartz Sand [%] *
OPC	100	0	0	6	94
LiN	100	0	0.74	6	94
MK-5	95	5	0	6	94
MK-10	90	10	0	6	94
MK-15	85	15	0	6	94
MK-20	80	20	0	6	94
MK-5-LiN	95	5	0.74	6	94
MK-10-LiN	90	10	0.74	6	94
MK-15-LiN	85	15	0.74	6	94
MK-20-LiN	80	20	0.74	6	94

* A total of 100% of quartz sand in mortars used for the strength activity index.

**Table 3:** Reduction of mortars expansion after 14 and 28 days, as against the control mortar.

Sample Code	Time [Days]	
	14 Days	28 Days
	Expansion Reduction [%]	
LiN	30.96	36.94
MK-5	17.81	17.65
MK-10	70.41	62.35
MK-15	75.89	69.41
MK-20	92.33	89.41
MK-5-LiN *	58.36	68.24
MK-10-LiN	76.99	52.94
MK-15-LiN	89.59	83.53
MK-20-LiN	97.26	97.65

* mortar degraded.

**Table 4:** Composition of potassium-sodium-calcium silicate gels obtained in the EDS analysis.

Sample Code	Point	Location	Chemical Composition [Mass %]					Ratio of Mass Percent	
			Si	Ca	Al	Na	K	Ca/Si	(Na + K)/Si
OPC	1	aggregate	64.2	11.4	-	18.2	2.8	0.18	0.33
	2	ITZ	66.2	18.1	0.9	11.9	2.5	0.27	0.22
LiN	1	aggregate	76.3	3.5	0	8.6	0	0.05	0.11
	2	rim	54.1	27.6	0.5	9.4	0.6	0.51	0.18
	3	paste	39.3	34.4	3.1	5.1	0.4	0.87	0.14
MK-5	1	ITZ	58.0	31.6	0.8	5.5	0.8	0.54	0.11
	2	aggregate	75.5	13.9	1.1	4.8	0.6	0.18	0.07
	3	paste	51.7	30.9	1.4	9.0	0.7	0.60	0.19
MK-10	1	aggregate	81.9	3.8	0	8.1	6.2	0.05	0.17
	2	aggregate	82.6	4.6	0.7	6.9	4.0	0.06	0.13
	3	paste	75.7	7.5	0.8	9.1	2.5	0.10	0.15
	4	ITZ	85.5	3.15	0.5	10.4	2.1	0.04	0.15
MK-5 LiN	1	aggregate	94.09	1.65	0.65	2.85	0.77	0.02	0.04
	2	ITZ	50.9	29.8	1.7	8.6	0.7	0.59	0.18
	3	ITZ	61.8	24.3	2.0	3.6	0.0	0.39	0.06
	4	paste	48.8	36.7	3.6	3.1	0.0	0.75	0.06
MK-10 LiN	1	rim	62.1	18.8	0.7	9.8	2.2	0.30	0.19
	2	aggregate	75.7	4.2	0.7	12.2	0.7	0.06	0.17
	3	paste	72.5	8.5	0.76	10.8	1.9	0.12	0.17

## Data Availability

Not applicable.

## References

[1] Stanton T.E. (1940). Expansion of concrete through reaction between cement and aggregate. Proc. Am. Soc. Civ. Eng..

[2] Garcia-Diaz E., Riche J., Bulteel D., Vernet C. (2006). Mechanism of damage for the alkali-silica reaction. Cem. Concr. Res..

[3] Struble L., Diamond S. (1981). Unstable swelling behaviour of alkali silica gels. Cem. Concr. Res..

[4] Stark J., Freybur E., Seyfarth K., Giebson C. (2006). AKR-PrüfverfahrenzurBeurteilung von Gesteinskörnungen und project spezifischen Betonen. Beton.

[5] Wen J., Dong J., Chang C., Xiao X., Zheng W. (2022). Alkali−Silica Activity and Inhibition Measures of Concrete Aggregate in Northwest China. Crystals.

[6] Prin D., Brouxel M. Alkali-aggregate reaction in nortiiern France: A review. Proceedings of the 9th International Conference on Alkali-Aggregate Reaction in Concrete.

[7] Lukschová Š. (2009). Alkali-Silica Reaction of Aggregates in Real Concrete and Mortar Specimen. Ph.D. Thesis.

[8] Sims I., Poole A.B. (2017). Alkali-aggregate reaction in concrete: A world review.

[9] Bakker J. Control of ASR related risks in the Netherlands. Proceedings of the 13th International Conference on Alkali-Aggregate Reaction in Concrete.

[10] Tcherner J., Aziz T. Effects of AAR on seismic assessment of nuclear power plants for life extensions. Proceedings of the 20th International Conference on Structural Mechanics in Reactor Technology (SMiRT).

[11] Oberholster R.E., Wan Aardt J.H.P., Brandt M.P. (1983). Structure and Performance of Cements, (red. P. Barnes). Appl. Sci. Publ..

[12] Rajabipour F., Giannini E., Dunant C., Ideker J.H., Thomas M.D.A. (2015). Alkali–silica reaction: Current understanding of the reaction mechanisms and the knowledge gaps. Cem. Concr. Res..

[13] Thomas M.D.A., Folliard K.J., Page C.L., Page M.M. (2007). Concrete aggregates and the durability of concrete. Durability of Concrete and Cement Composites.

[14] Shehata M., Thomas M.D.A., Bleszynski R.F. (1999). The effect of fly composition on the chemistry of pore solution. Cem. Concr. Res..

[15] Shehata M.H., Thomas M.D.A. (2002). Use of ternary blends containing silica fume and fly ash to suppress expansion due to alkali-silica reaction in concrete. Cem. Concr. Res..

[16] Bleszynski R.F. (2002). The Performance and Durability of Concrete with Ternary Blends of Silica Fume and Blast-Furnace Slag. Ph.D. Thesis.

[17] Ramlochan T., Thomas M.D.A., Gruber K.A. (2000). The effect of metakaolin on alkali silica reaction in concrete. Cem. Concr. Res..

[18] Thomas M. (2011). The effect of supplementary cementing materials on alkali-silica reaction: A review. Cem. Concr. Res..

[19] Menéndez E., Sanjuán M.Á., García-Roves R., Argiz C., Recino H. (2020). Sustainable and Durable Performance of Pozzolanic Additions to Prevent Alkali-Silica Reaction (ASR) Promoted by Aggregates with Different Reaction Rates. Appl. Sci..

[20] Czapik P. (2018). Degradation of Glaukonite Sandstone as a Result of Alkali-Silica Reactions in Cement Mortar. Materials.

[21] Wei S., Zheng K., Zhou J., Prateek G., Yuan Q. (2022). The combined effect of alkalis and aluminium in poresolution on alkali-silica reaction. Cem. Concr. Res..

[22] Chatterji S. (1983). The role of Ca(OH)_2_ in the breakdown of Portland cement concrete due to alkali-silica reaction. Cem. Concr. Res..

[23] Vollpracht A., Lothenbach B., Snellings R., Haufe J. (2016). The pore solution of blended cements: A review. Mater. Struct..

[24] Chappex T., Scrivener K.L. (2013). The Effect of Aluminum in Solution on the Dissolution of Amorphous Silica and its Relation to Cementitious Systems. J. Am. Ceram. Soc..

[25] Bickmore B.R., Nagy K.L., Gray A.K., Brinkerhoff A.R. (2006). The effect of Al(OH)_4_ on the dissolution rate of quarto. Geochim. Cosmochim. Acta.

[26] Houston J.R., Herberg J.L., Maxwell R.S., Carroll S.A. (2008). Association of dissolved aluminum with silica: Connecting molecular structure to surface reactivity using NMR. Geochim. Cosmochim. Acta.

[27] McCoy W.J., Caldwell A.G. (1951). New approach in inhibiting alkali–aggregate expansion. ACI Mater. J..

[28] Demir İ., Arslan M. (2013). The Mechanical and Microstructural Properties of Li_2_SO_4_, LiNO_3_, Li_2_CO_3_ and LiBr Added Mortars Exposed to Alkali-Silica Reaction. Constr. Build. Mater..

[29] Thomas M.D.A., Fournier B., Folliard K.J., Ideker J.H., Resendez Y. (2007). The Use of Lithium to Prevent or Mitigate Alkali-Silica Reaction in Concrete Pavements and Structures.

[30] Thomas M.D.A., Hooper T., Stokes D. Use of lithium-containing compounds to control expansion in concrete due to ASR. Proceedings of the 11 International Conference on Alkali-Aggregate Reaction.

[31] Feng X. (2008). Effects and Mechanisms of Lithium Nitrate on Controlling Alkali-Silica Reaction. Ph.D. Thesis.

[32] Zapała-Sławeta J., Owsiak Z. (2016). The role of lithium compounds in mitigating alkali-gravel aggregate reaction. Constr. Build. Mater..

[33] Kawamura M., Fuwa H. (2003). Effects of lithium salts on ASR gel composition and expansion of mortars. Cem. Concr. Res..

[34] Leemann A., Lörtscher L., Bernard L., Le Saout G., Lothenbach B., Espinosa-Marzal R.M. (2014). Mitigation of ASR by the use of LiNO3—Characterization of the reaction products. Cem. Concr. Res..

[35] Tremblay C., Bérubé M.A., Fournier B., Thomas M.D., Folliard K.J. (2007). Effectiveness of lithium-based products in concrete made with Canadian natural aggregates susceptible to alkali–silica reactivity. ACI Mater. J..

[36] Afshinnia K., Rangaraju R.P. (2015). Efficiency of ternary blends containing fine glass powder in mitigating alkali-silica reaction. Constr. Build. Mater..

[37] Moser R.D., Jayapalan A.R., Garas V.Y., Kurtis K.E. (2010). Assessment of binary and ternary blends of metakaolin and Class C fly ash for alkali–silica reaction mitigation in concrete. Cem. Concr. Res..

[38] Chappex T. (2012). The Role of Aluminium from Supplementary Cementitious Materials in Controlling Alkali-Silica Reaction. Ph.D. Thesis.

[39] Drimalas T., Ideker J.H., Bentivegna A.F., Folliard K.J., Fournier B., Thomas M.D.A. (2012). The long-term monitoring of large-scale concrete specimens containing lithium salts to mitigate alkali–silica reaction. Spec. Publ..

[40] Venkatanarayanan H.K., Rangaraju P.R. (2014). Effectiveness of Lithium Nitrate in Mitigating Alkali-Silica Reaction in the Presence of Fly Ashes of Varying Chemical Compositions. J. Mater. Civ. Eng..

[41] Zapała-Sławeta J. (2017). Influence of Exposure Conditions on the Efficacy of Lithium Nitrate in Mitigating Alkali Silica Reaction. IOP Conf. Ser. Mater. Sci. Eng..

[42] Zapała-Sławeta J., Owsiak Z. (2017). Alkali Silica Reaction in the Presence Of Metakaolin—The Significant Role of Calcium Hydroxide. IOP Conf. Ser. Mater. Sci. Eng..

[43] Chen J.J., Li Q.H., Ng P.L., Li L.G., Kwan A.K.H. (2020). Cement Equivalence of Metakaolin for Workability, Cohesiveness. Strength and Sorptivity of Concrete. Materials.

[44] Justice J.M. (2005). Evaluation of Metakaolins for Use as Supplementary Cementitious Materials. Master’s Thesis.

[45] Özcan F., Kaymak H. (2018). Utilization of Metakaolin and Calcite: Working Reversely in Workability Aspect—As Mineral Admixture in Self-Compacting Concrete. Hindawi Adv. Civ. Eng..

[46] (2014). Standard Test Method for Potential Alkali Reactivity of Aggregates (Mortar-Bar Method).

[47] (2013). Standard Test Method for Determining the Potential Alkali-Silica Reactivity of Combinations of Cementitious Materials and Aggregate (Accelerated Mortar Bar Method).

[48] Hoobs D.V. (1988). Alkali-Silica Reaction in Concrete.

[49] Owsiak Z. (2007). Testing alkali-reactivity of selected concrete aggregates. J. Civ. Eng. Manag..

[50] (2017). Methods of Testing Cement—Part 1: Determination of Strength.

[51] (2014). Fly Ash for Concrete—Part 1: Definition, Specifications and Conformity Criteria.

[52] (2020). Standard Practice for Mechanical Mixing of Hydraulic Cement Pastes and Mortars of Plastic Consistency.

[53] Aliyu I., Lawan A., Kaura J.M., Ozovehe A.A. (2020). Pozzolanic Strength Activity Index of Metakaolin Processed from Kankara Kaolin According to BS EN 450-1. J. Sci. Educ. Technol...

[54] Pavlíková M., Brtník T., Keppert M., Černý R. (2009). Effect of metakaolin as partial Portland-cement replacement on properties of high performance mortars. Cem. Lime Concr..

[55] Liu H., Wang Z., Tian Z., Bu J., Qiu J. (2022). Effect and Mechanism of Metakaolin Powder (MP) on Rheological and Mechanical Properties of Cementitious Suspension. Materials.

[56] Wang W.C. (2014). Effects of fly ash and lithium compounds on the water soluble alkali and lithium content of cement specimens. Constr. Build. Mater..

[57] Shin J.-H., Struble, Kirkpatrick R.J. (2015). Microstructural Changes Due to Alkali-Silica Reaction during Standard Mortar Test. Materials.

[58] Zapała-Sławeta J. (2017). The effect of meta-halloysite on alkali–aggregate reaction in concrete. Mater. Struct..

[59] Diamond S. Alkali aggregate reactions in concrete pore-solution effects. Proceedings of the 6th International Conference of Alkalis in Concrete.

[60] Leemann A., Bernard L., Alahrache S., Winnefeld F. (2015). ASR prevention—Effect of aluminum and lithium ions on the reaction products. Cem. Concr. Res..

